# Sex‐Specific Regulation of the Turandot Gene Family Modulates Temperature‐Dependent Lifespan in *Drosophila melanogaster*

**DOI:** 10.1111/acel.70564

**Published:** 2026-05-29

**Authors:** Jessica M. Hoffman, A. Tate Lasher, Michael P. Fitch, Steven N. Austad, Liou Y. Sun

**Affiliations:** ^1^ Department of Biology University of Alabama at Birmingham Birmingham Alabama USA; ^2^ Department of Biological Sciences Augusta University Augusta Georgia USA

## Abstract

Ambient temperature is a primordial determinant of longevity across the animal kingdom, yet the molecular transducers that couple thermal cues to aging rates remain elusive. Here, we interrogate the transcriptomic and metabolomic landscapes of *Drosophila melanogaster* to decode the mechanisms of temperature‐dependent lifespan extension. We find that thermal stress drives a profound remodeling of the transcriptome that surprisingly outpaces metabolic adaptation. Through this multi‐omics integration, we identify the Turandot (*tot*) gene family as a significant factor in the thermal longevity response. Intriguingly, *tot* genes are not merely passive responders to temperature; rather, they actively regulate lifespan. We demonstrate that RNAi‐mediated knockdown of *tot* expression is sufficient to extend longevity across a range of temperatures, uncoupling the survival benefit from the thermal environment. Furthermore, this extension exhibits distinct sexual dimorphism, suggesting a sex‐specific strategy in stress‐response allocation. These findings establish the Turandot family as a novel, genetically separable regulator of aging, challenging the view that temperature‐mediated longevity is solely a passive thermodynamic consequence.

## Graphical abstract

Multi‐omics analysis in *Drosophila* reveals that temperature‐driven lifespan changes are actively regulated, not purely thermodynamic. The Turandot gene family mediates this response, and its knockdown extends lifespan across temperatures with sex‐specific effects, establishing tot genes as key genetic regulators of thermal longevity.

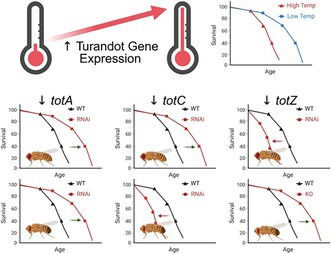

## 1 Introduction

Over a century ago, temperature was shown to be a major factor controlling lifespan. When flies were placed in progressively lower temperatures, they lived progressively longer (Loeb and Northrop 1916). Since then, the inverse relationship between temperature and longevity has been robustly replicated. Almost invariably, decreasing temperature to a non‐lethal degree increases longevity across the animal kingdom (reviewed in Keil et al. 2015). For example, fruit flies, *Drosophila subobscura* (Smith 1958) and *D. melanogaster* (Miquel et al. 1976), house flies (Ragland and Sohal 1975), and worms (Klass 1977) raised at lower temperatures have increased lifespan. Similar effects have been reported in poikilothermic vertebrates (e.g., Valenzano et al. 2006; Walford and Liu 1965). Even in endotherms, interventions that reduce body temperature have been associated with lifespan extension (Conti et al. 2006).

In humans, lower body temperatures have been associated with extended survival. Participants in the Baltimore Longitudinal Study of Aging in the lower 50% of body temperature outlived those in the upper 50% (Roth et al. 2002) and analysis of large cross sectional data has indicated that lower body temperature is associated with advanced age (Waalen and Buxbaum 2011). Further, calorie restriction interventions have been shown to reduce the body temperature of both nonhuman primates (Lane et al. 1996) and humans (Soare et al. 2011). Work in rodents shows that preventing body temperature reductions (i.e., by housing at thermoneutrality) ameliorates the effect that lower calorie intake has on lifespan (Koizumi et al. 1996; Zhao et al. 2022). The robustness of the negative correlation between lifespan and temperature coupled with the observations of reduced body temperature in long‐lived humans has prompted significant interest in elucidating the molecular mechanisms involved in this relationship.

Two of the most frequently proposed explanations for lifespan extension at lower temperatures are (1) reduced metabolic rate and (2) decreased oxidative stress. These mechanisms are closely related and align with the “rate of living” theory, which posits that higher metabolic rates and oxygen consumption shorten lifespan (Pearl 1928). Higher temperatures have been shown to increase metabolic rates in numerous species (reviewed in Hou and Amunugama 2015). In addition, fruit flies (MacMillan et al. 2016) and carp (Gracey et al. 2004) exposed to cold temperatures had significant changes in free radical metabolism. Lipid peroxidation, a measure of free radical activity, is also higher in fruit flies raised at warm temperatures (Sestini et al. 1991). Important limitations exist with these explanations, however. Isolated mitochondria display increases in the production of reactive oxygen species, a source of oxidative stress, following moderate reductions in temperature (Ali et al. 2010). Additionally, rodents housed at their thermoneutral zone (with resultant body temperature elevations) display reduced lifespans despite lower metabolic rates compared to room‐temperature housed controls. This short lifespan is rescued when body temperature is reduced to that of room‐temperature housed individuals (achieved by cage ventilation) while metabolic rate remains low (Zhao et al. 2022). These demonstrate that additional mechanisms, beyond reductions in metabolic rate or oxidative stress, drive lifespan extension at low temperatures.

Most of the recent work done on temperature and longevity has used the nematode *C. elegans* to explore molecular mechanisms underlying temperature‐dependent longevity. Thermosensory neurons have been shown to regulate *daf‐9* mRNA levels which subsequently alter DAF‐12 activity (Lee and Kenyon 2009) and thermosensitive TRP channels have been shown to mediate neuronal or intestinal calcium influx which subsequently alters DAF‐16 activity (Xiao et al. 2013) to modulate longevity responses to temperature. However, the mechanisms underlying similar effects in other species remain poorly characterized.

While the precise pathways by which mild temperature reduction extends lifespan are not yet fully understood, numerous studies have explored molecular responses to extreme temperature conditions. Fruit flies adapted to extreme cold temperatures (6°C) have significantly different transcriptomes and metabolomes compared to those maintained under standard conditions (MacMillan et al. 2016). In addition, a sizeable amount of work has looked at the molecular changes that occur during development and early life in response to temperature. For example, the metabolome is shaped by developmental as well as adult temperature (Hariharan et al. 2014), with each having different effects on overall metabolic network connectivity. Interestingly, fish reared at cooler temperatures have increased 20S proteasome activity, but no changes in mTOR, one of the most studied pathways that regulates aging and longevity (Lu and Hsu 2015). Conversely, work in flies suggests that 4E‐BP, a downstream mTOR effector protein, can partially regulate temperature dependent longevity (Carvalho et al. 2017). Overall, the long‐term effects of temperature exposure that lead to variation in longevity have yet to be fully determined.

Our aim with this study was to directly address gaps in our understanding of how temperature manipulation influences longevity by identifying genetic and molecular mechanisms for temperature‐dependent lifespan extension. To this end, we completed a transcriptomic and metabolomic analysis of adult *D. melanogaster* maintained at three different temperatures. Through this analysis we identified the *tot* family of genes as a novel mediator for temperature‐dependent lifespan extension with notable sex‐specific effects. Further, we identified numerous genetic pathways and metabolic alterations associated with temperature, sex, and their interaction, whose individual and combined effects may provide novel insights into environmental effects on lifespan.

## 2 Methods

### 2.1 Drosophila Husbandry

*D. melanogaster w* ^*1118*^ flies were procured from the Bloomington *Drosophila* Stock Center and were maintained in plastic vials at 24°C on a 12/12 light/dark cycle and ~60% humidity. Flies were fed a standard cornmeal (6%), dextrose (5.5%), sucrose (3%), and yeast (2.5%) diet with propionic acid (3 mL/L) added as an antifungal. 3 mL of diet was added to each vial. *tot* gene mutants were obtained from the Bloomington Stock Center, RNAi lines from the TRiP panel and a *totZ* knockout in the *yw* background. *totA* and *totC* RNAi lines were crossed to actin and daughterless UAS/gal4 drivers, respectively, received from Dr. Nicole Riddle (UAB). RNAi lines without the driver were used as controls.

### 2.2 Experimental Design

Flies were mated and eggs laid for 48 h. For TRiP RNAi lines, male driver lines were crossed to virgin female TRiP lines. Parents were discarded, and embryos were allowed to develop. Virgin male and female flies were collected within 8 h of eclosion and maintained at 24°C. At approximately 5 days of age, flies were randomized onto one of three temperature treatments (18°C, 24°C, or 28°C) in groups of approximately 100 flies per sex/temperature at a density of 20 flies per vial. Flies were transferred onto new media three times a week, and deaths were recorded. Transferring continued until all flies were dead.

### 2.3 Metabolomics and RNAseq Sample Collection

**FIGURE 1:**
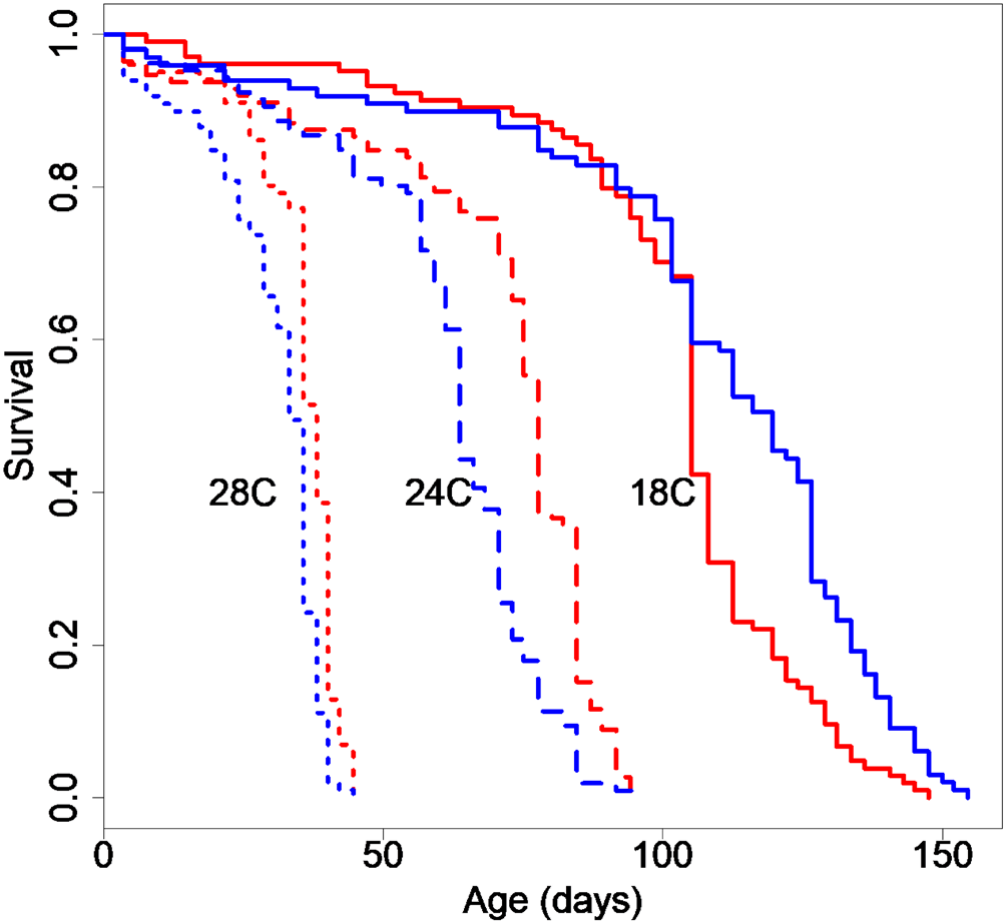
Kaplan Meier curves of temperature and sex for w^1118^ flies. Females are shown in red, males in blue. The temperatures that flies were maintained at as adults are shown next to their corresponding curves, each with a different line type. Our Cox proportional model indicates a significant effect of temperature, sex, and temperature‐by‐sex interaction (*p* < 5.62 × 10^−7^). For 28 degrees *N* = 101 females, 99 males; for 24 degrees *N* = 112 females, 106 males; for 18 degrees *N* = 104 females, 99 males.

Flies were collected as virgins in groups of 20 flies per vial, randomized to temperature treatment, and maintained as described above. When flies reached 45 days of age at 18°C and 24°C and 35 days at 28°C, they were flash frozen in liquid nitrogen in groups of 10. Forty‐five days of age is the point at which the 18°C and 24°C began to diverge on the survival curves (Figure 1), and thus likely represent biologically interesting and significant shifts in the aging process. However, the majority of flies at 28°C are dead by this point, so we chose the time point at which about 50% mortality had occurred. All collections took place between 10:30 and 12:00 hours. Flies were then stored at −80°C until sample processing.

### 2.4 Metabolomics

Metabolomics samples were randomized, such that temperatures would not be all run at the same time and then sent to the Emory University Clinical Biomarkers Laboratory for global metabolite profiling. Methods for metabolomics data acquisition have been previously published (Hoffman et al. 2019; Liu et al. 2016; Soltow et al. 2013). Briefly, frozen samples were pulverized, treated with 100 μL acetonitrile, and incubated for 30 min on ice. Samples were then centrifuged (16,100 × *g* at 4°C) for 10 min. Pooled plasma (Qstd3) was processed and run alongside samples, and an aliquot of National Institute of Standards and Technology Standard Reference Material 1950 (NIST SRM1950) was processed and analyzed identically to the samples prior to the first run and after the final run. The resulting supernatant was collected and transferred to new vials.

Samples were then analyzed with liquid chromatography coupled to Fourier transform high‐resolution mass spectrometry. Samples were run in triplicate on a Dionex Ultimate 3000, Orbitrap Fusion Tribrid Mass Spectrometer system (Thermo Scientific) operated at 120,000 resolution. All replicates were analyzed using hydrophilic interaction liquid chromatography (HILIC) with electrospray ionization (ESI) source operated in positive mode and reverse‐phase chromatography (RPC) with ESI operated in negative mode. Analyte separation for HILIC was accomplished by a 2.1 mm × 50 mm × 2.5 μm Waters XBridge BEH Amide XP HILIC and an eluent gradient (A = water, B = acetonitrile, C = 2% formic acid) consisting of an initial 1.5‐min period of 22.5% A, 75% B, and 2.5% C followed by a linear increase to 77.5% A, 20% B, and 2.5% C at 4 min and a final hold of 1 min. RPC separation was by 2.1 mm × 50 mm × 3 μm end‐capped C18 column (Higgins) using an eluent gradient (A = water, B = acetonitrile, C = 10 mM ammonium acetate) consisting of an initial 1‐min period of 60% A, 35% B, and 5% C, followed by a linear increase to 0% A, 95% B, and 5% C at 1.5 min and held for the remaining 3.5 min. The mobile phase flow rate for HILICpos was held at 0.350 mL/min for the first 1.5 min, and increased to 0.400 mL/min for the remaining of the run. C18neg mobile phase flow rate was held at 0.400 mL/min for the first 2 min and then increased to 0.500 mL/min for the remaining 3.0 min. Data were collected for a mass‐to‐charge ratio (m/z) range 85–1275. Probe temperature, capillary temperature, sweep gas, and S‐Lens RF levels were maintained at 200°C, 300°C, 1 arbitrary units (AU), and 45, respectively, for both ESI polarities. Additional source tune settings were optimized for sensitivity using a standard mixture; positive tune settings for sheath gas, auxiliary gas, sweep gas, and spray voltage setting were 45 AU, 25 AU, 1 AU, and 3.5 kV, respectively; negative settings were 45 AU, 5 AU, 1 AU, and −3.0 kV. Maximum C‐trap injection times of 100 ms and automatic gain control target of 1 × 106 for both polarities. During untargeted data acquisition, no exclusion or inclusion masses were selected, and data were acquired in MS1 mode only. Data were stored as .raw files and converted to CDF format using Xcalibur file converter software (Thermo Fisher, San Diego, CA) for further data processing. Peak detection, noise filtering, m/z and retention time alignment, feature quantification, and data quality filtering were performed using apLCMS (Yu et al. 2009) with xMSanalyzer (Uppal et al. 2013). Data were extracted as m/z features where a feature is defined by m/z, retention time, and integrated ion intensity.

### 2.5 RNA Sequencing

Samples were shipped on dry ice to BGI (Shenzhen, China) for transcriptomic profiling. Briefly, RNA was extracted from each fly sample using TRIzol and DNase I treated. 200 ng RNA passing quality control (RIN > 7; Agilent 2100 Bio analyzer) was submitted for poly‐A selection and 100 bp paired‐end sequencing on the BGI BGISEQ‐500 platform. Reads were then first analyzed in BGI internal software SOAPnuke to filter and remove low quality reads. HISAT (Kim et al. 2015) was then used to map reads onto the *Drosophila* genome, and StringTie (Pertea et al. 2015) with Cuffcompare (Trapnell et al. 2012) were used to compare transcripts to known annotated genes on the *Drosophila* genome. Novel transcripts were predicted with CPC (Kong et al. 2007), and rMATS was used to determine differentially spliced genes. Lastly, gene expression levels were determined by mapping clean reads to the Drosophila reference using Bowtie2 (Langmead and Salzberg 2012) and calculated expected counts with RSEM (Li and Dewey 2011). These final expected expression counts were used for statistical analysis below. The RNA sequencing data has been deposited at the Gene Expression Omnibus and are publicly accessible (Accession: GSE229837).

### 2.6 Statistical Analyses

All analyses were completed in the program R unless otherwise stated (R Core Team 2026). Our final dataset consisted of 24 metabolomics samples (4 per sex/temperature, each sample with 10 flies) and 23 transcriptomics samples (4 per sex/temperature, with male 28°C having only 3). Our final metabolomic and transcriptomic datasets consisted of 3071 metabolites in the positive ion mode, 3129 metabolites in the negative ion mode, and 9698 genes in our transcriptomic assay.

*Longevity*: Cox proportional hazard models were used to determine significant differences in sex and temperature survival. Survival curves were created with Kaplan–Meier plots.

*Metabolomics*: We analyzed the resulting analytes from the AE and HILIC column separately. First, metabolomics data were log transformed, centered, and scaled. Then we were interested in determining which individual metabolites were associated with temperature, sex, and their interaction. To this end, we ran a linear model of the three variables on each metabolite, controlling for multiple comparisons using a False Discovery Rate (FDR) of *α* < 0.05. For those metabolites found to be significantly associated with one of our variables of interest, we ran them through the metabolic enrichment program mummichog (Li et al. 2013). In addition, we ran unsupervised principal components analysis (PCA) to determine the degree to which the entire metabolome was associated with temperature and sex.

Transcriptomics: Raw expected gene counts were normalized using the “cpm” function in edgeR (Robinson et al. 2010), and any genes that had normalized gene counts less than 0.5 were removed from the analysis. We first ran a general linear model looking at the effects of sex, temperature, and their interaction on rounded gene counts using a Poisson distribution. Significance of genes was set at an FDR of *α* < 0.01. Genes that were found to be significantly associated with our factors of interest were run through gene ontology (GO) analysis using the goseq package (Young et al. 2010). We then narrowed down our GO results by only looking at those categories that had 10–50 genes in the category. This removed all small categories that are easily inflated by one overexpressed gene and the large categories that are overly vague. GOs were considered to be significantly overrepresented at an FDR *α* < 0.05. Where group comparisons were made for visualization of transcriptomic changes, rounded expected gene counts between indicated groups were compared in edgeR using a Poisson distribution and were visualized using the EnhancedVolcano package. Similar to our metabolomics assay, we ran unsupervised PCA to determine if the entire metabolome was associated with our factors of interest. We finally looked at transcript and sample relatedness visually using heatmap generation.

## 3 Results

### 3.1 Transcriptomic Changes of Temperature‐Dependent Lifespan

As expected, cooler temperature led to a significantly longer lifespan in both males and females (Figure 1). We found a significant effect of sex, temperature, and their interaction in our Cox proportional hazard model (Table 1). Females were longer lived than males at the two warmer temperatures, and males were longer lived than females at the coolest temperature.

**TABLE 1:** Cox proportional hazard results.

	coef	exp(coef)	se(coef)	*z*	Pr(>|*z*|)
Sex–Male	−2.52478	0.08008	0.50453	−5.004	5.61E‐07
Temperature	0.47938	1.61508	0.02685	17.853	2.72E‐71
Sex × Temperature interaction	0.1177	1.1249	0.0213	5.527	3.26E‐08

We were first interested in determining individual genes and genetic pathways that might be implicated in the extended longevity phenotype seen at lower temperatures. We found more transcripts associated with sex (38%) as compared to temperature (30%) in our gene expression analysis. Our interaction analysis also discovered that about 16% of the individual genes analyzed had a temperature by sex interaction (Table 2, Table S1).

**TABLE 2:** Cox proportional hazard results for *tot* mutant fly lifespan experiments.

Mutant	Effect	18°C			24°C			28°C		
		HR	CI	*p*	HR	CI	*p*	HR	CI	*p*
*totA*	RNAi (vs. Control)	0.1709	0.12–0.24	< 0.0001	0.6631	0.49–0.90	0.0086	0.3128	0.22–0.44	< 0.0001
	Male (vs. Female)	3.5969	2.61–4.96	< 0.0001	1.3972	1.04–1.88	0.0259	4.5714	3.33–6.26	< 0.0001
	Interaction	0.6323	0.40–0.99	0.0442	0.3729	0.25–0.57	< 0.0001	0.7698	0.50–1.18	ns
*totC*	RNAi (vs. Control)	0.2551	0.18–0.36	< 0.0001	0.7209	0.52–1.00	0.0514	0.0536	0.03–0.09	< 0.0001
	Male (vs. Female)	1.5998	1.20–2.14	0.0015	1.4100	1.04–1.91	0.0268	3.000	2.23–4.04	< 0.0001
	Interaction	7.2553	4.62–11.4	< 0.0001	4.2328	2.71–6.60	< 0.0001	52.432	27.4–100.1	< 0.0001
*totZ*	Knockout (vs. Control)	6.0287	4.15–8.76	< 0.0001	5.6041	3.99–7.87	< 0.0001	8.046	5.45–11.9	< 0.0001
	Male (vs. Female)	8.0498	5.43–11.9	< 0.0001	4.9832	3.58–6.94	< 0.0001	13.288	8.89–19.8	< 0.0001
	Interaction	0.0761	0.05–0.13	< 0.0001	0.1968	0.13–0.30	< 0.0001	0.0674	0.04–0.11	< 0.0001

Abbreviations: CI, 95% confidence interval (lower–upper); HR, hazard ratio; *p*, the *p*‐value for the indicated effect.

### 3.2 Effect of Turandot Knockdown on Lifespan

**FIGURE 2:**
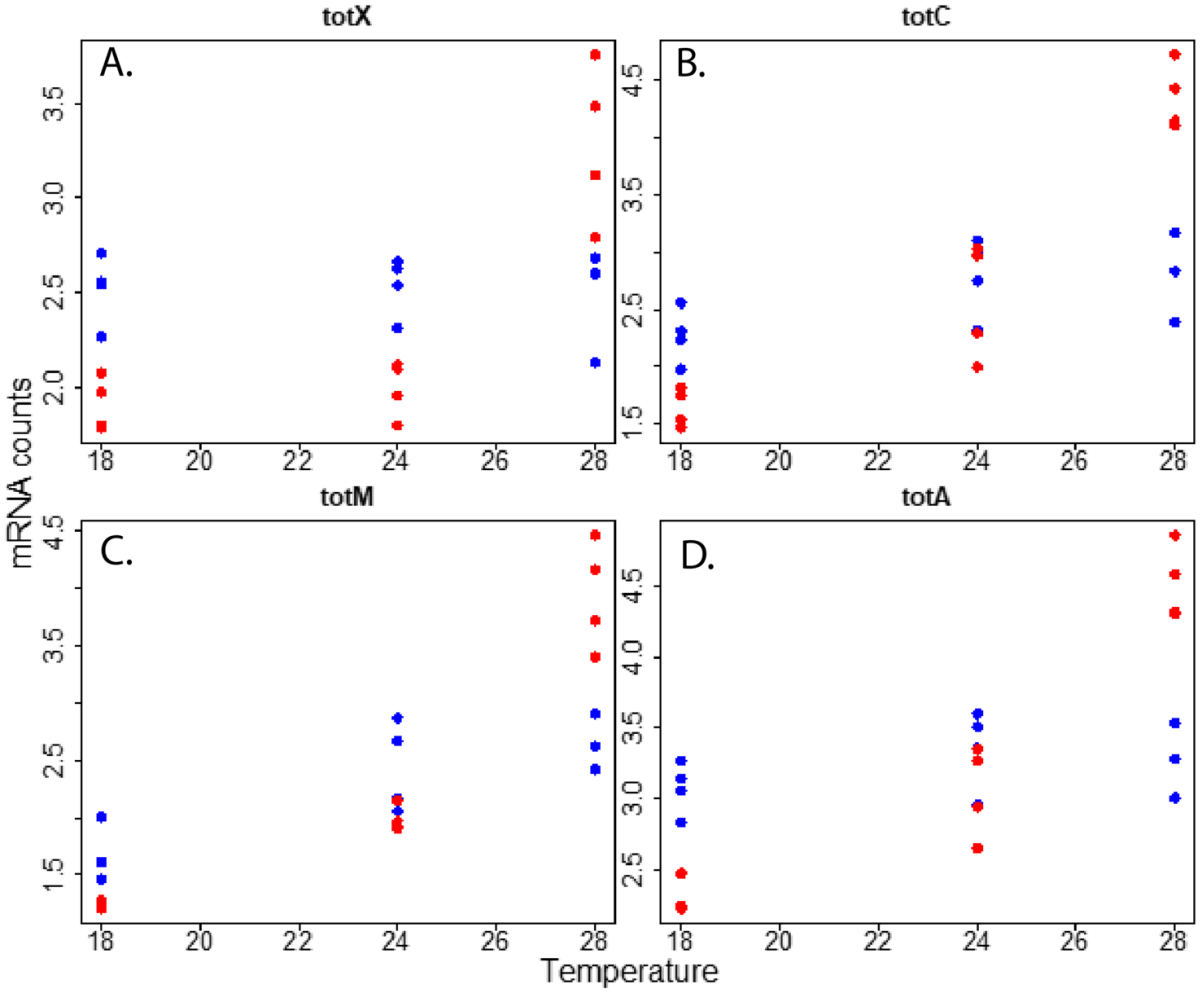
*tot* gene expression from RNAseq data. Log transformed counts of *totX* (A), *totC* (B), *totM* (C), and *totA* (D) are shown for visual purposes. Each dot represents a sample, females are shown in red, and males in blue. For all genes there was a significant temperature, sex, and sex‐by‐temperature interaction.

**FIGURE 3:**
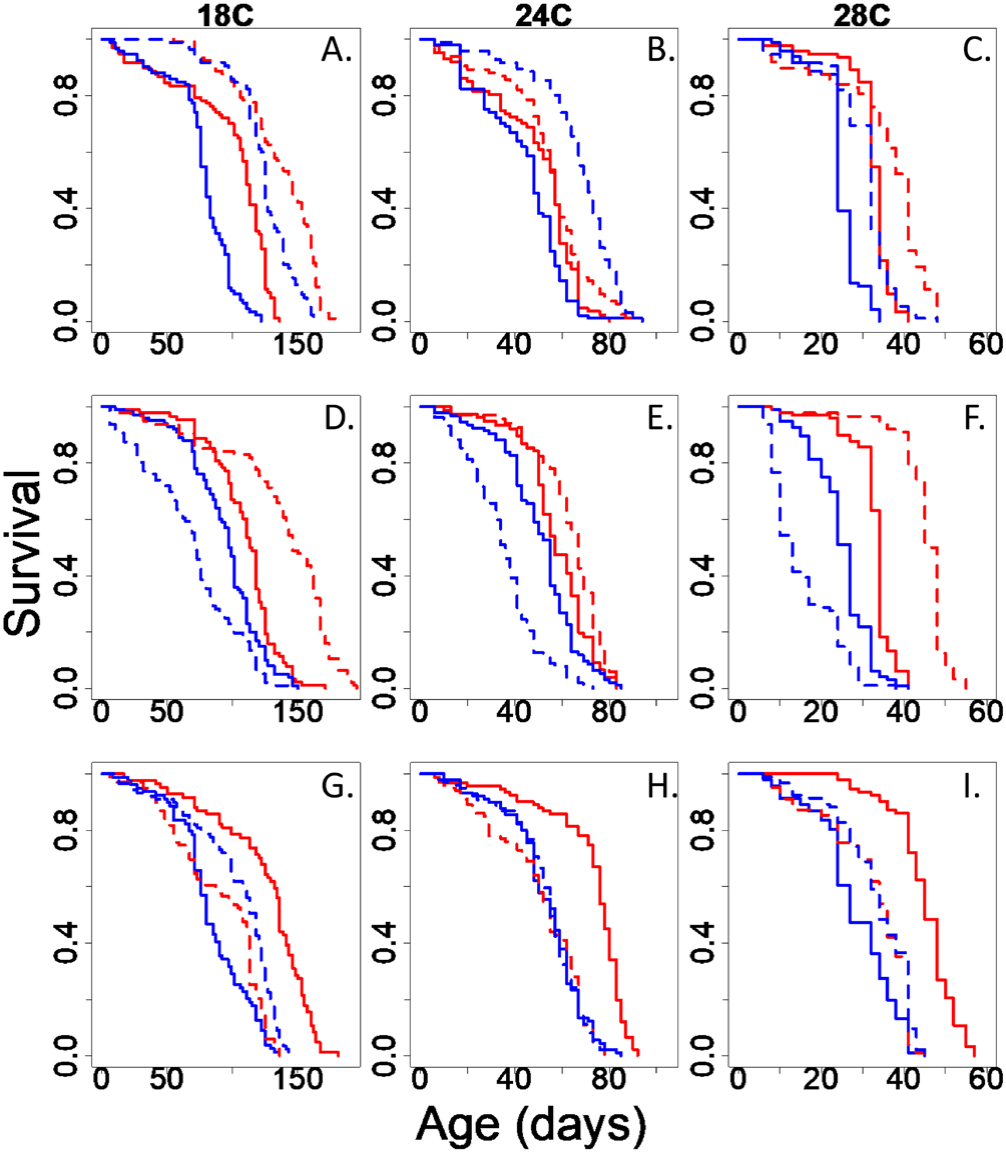
Longevity of three different tot genes at three different temperatures. Solid lines are controls, dashed lines are genetically modified. Males in blue, females in red. *totA* RNAi knockdown increased lifespan in males at all temperature and females at 18° and 28° (A–C). *totC* knockdown increased lifespan in females at all temperatures and decreased lifespan in males at all temperatures (D–F). *totZ* knockout increased lifespan in males at 18° and 28° and decreased lifespan in females at all temperatures (G–I). Note the *totZ* knockout was maintained on a different genetic background that the two knockdowns. For *totA N* = 84–107; for *totC N* = 68–102; for *totZ N* = 79–102.

One set of genes that were consistently upregulated at higher temperatures and displayed markedly lower expression in our longest‐lived flies (Figure S2) were the Turandot (*tot*) genes (Figure 2). We then were interested in determining if knockdown of these genes led to longer lifespan at warm temperature. We tested the effects of knockdown of 3 easily obtainable lines (*totA, totC*, and *totZ*). Knockdown of *totA* led to an increase in lifespan in both sexes at all temperatures (Figure 3A–C). Cox proportional hazard modeling of *totA* knockdown lifespan data revealed the interaction of genotype by sex significantly lowered hazard ratios at 18°C and 24°C, indicating that the *totA* knockdown reduces hazard ratios more dramatically in males at these temperatures. At 28°C, the main effect of *totA* knockdown significantly reduced hazard ratio, and male sex significantly elevated hazard ratio, but their interaction was insignificant (Table 2). *totC* knockdown led to increased lifespan in females and shortened lifespan in males (Figure 3D–F). Cox proportional hazard modeling of *totC* knockdown lifespan data revealed the genotype by sex interaction significantly elevated hazard ratios at all temperatures, indicating *totC* knockdown significantly elevates hazard ratios in males compared to females (Table 2). *totZ* knockout led to increased lifespan in males and shortened lifespan in females (Figure 3G–I). Cox proportional hazard modeling of *totZ* knockout lifespan data revealed the genotype by sex interaction significantly reduced hazard ratios, indicating *totZ* knockout significantly reduces male hazard ratios compared to females (Table 2). Overall these lifespan effects were consistent across all temperatures, not just the warmest, when *tot* gene expression was the highest (Figure 2). It should however be noted that *totZ* flies were maintained on a different genetic background than *totA* or *totC* and thus, comparisons of effect size between mutations should be made with caution.

To more completely analyze the effect Turandot gene mutation has on temperature‐dependent lifespan regulation, we carried out cox proportional hazard modeling on the effect of increasing temperature, Turandot genotype, and their interaction stratified by sex. In our *totA* cohort for both females and males, we detected a significant increase in hazard ratio with temperature elevation, a significant reduction in hazard ratio from *totA* RNAi, and a significant increase in hazard ratio from the temperature by RNAi interaction effect (Table 3). In our *totC* cohort for both females and males, we detected a significant increase in hazard ratio with temperature elevation; however, the effects of *totC* RNAi and the interaction of temperature by RNAi were insignificant (Table 3). In our female *totZ* cohort, we detected a significant increase in hazard ratio with increasing temperature or with *totZ* knockout, and a significant reduction of hazard ratio from the interaction of temperature elevation and *totZ* knockout (Table 3). In our male *totZ* cohort, we detected a significant increase in hazard ratio with increasing temperature, a significant reduction in hazard ratio from *totZ* knockout, and a significant elevation of hazard ratio from the interaction of temperature elevation and *totZ* knockout (Table 3).

**TABLE 3:** Cox proportional hazard results for the effects of temperature, Turandot genotype, and their interaction stratified by sex.

Mutant	Effect	HR	CI	*p*
*♀ totA*	Temperature	1.6345	1.55–1.73	< 0.0001
	RNAi (vs. Control)	0.0560	0.02–0.17	< 0.0001
	Interaction	1.0885	1.04–1.14	0.0041
*♂ totA*	Temperature	1.5713	1.50–1.65	< 0.0001
	RNAi (vs. Control)	0.0029	0.0007–0.01	< 0.0001
	Interaction	1.1977	1.13–1.26	< 0.0001
*♀ totC*	Temperature	1.8878	1.76–2.02	< 0.0001
	RNAi (vs. Control)	0.4812	0.15–1.58	ns
	Interaction	0.9813	0.93–1.03	ns
*♂ totC*	Temperature	1.4833	1.42–1.55	< 0.0001
	RNAi (vs. Control)	1.3595	0.50–3.67	ns
	Interaction	1.0203	0.98–1.06	ns
*♀ totZ*	Temperature	1.6769	1.59–1.77	< 0.0001
	Knockout (vs. Control)	14.213	4.20–48.0	< 0.0001
	Interaction	0.9497	0.90–1.0	0.0434
*♂ totZ*	Temperature	1.5263	1.45–1.60	< 0.0001
	Knockout (vs. Control)	0.1898	0.06–0.57	0.0032
	Interaction	1.0566	1.01–1.11	0.0192

Abbreviations: CI, 95% confidence interval (lower–upper); HR, hazard ratio; *p*, the *p*‐value for the indicated effect.

### 3.3 Influence of Temperature on “Aging” Genes

We also found significant effects of temperature on some, but not all, of the commonly studied longevity genes including InR, S6 kinase, and ribosomal protein S6 (Figure S1). Many of these genes had their highest expression at the lowest temperatures where flies lived the longest (Figure S1D,E), opposite what would potentially be expected. The expression of these “aging” genes was also generally higher in female flies, the generally longer‐lived group, compared to the male flies. We also found variation in how the Hsp's responded to temperature (Figure S3). Similar to the “aging” genes, many Hsp's were higher at the lower temperatures (Figure S3B–E), not necessarily the warmer temperatures, and several showed no significant changes with temperature (Figure S3H–K). When examined individually many of the Hsp's display clear differences in expression between the sexes, however the direction of this difference is inconsistent and a clear pattern (i.e., generally higher expression in one sex) is absent.

### 3.4 Metabolic Changes of Temperature‐Dependent Lifespan

**FIGURE 4:**
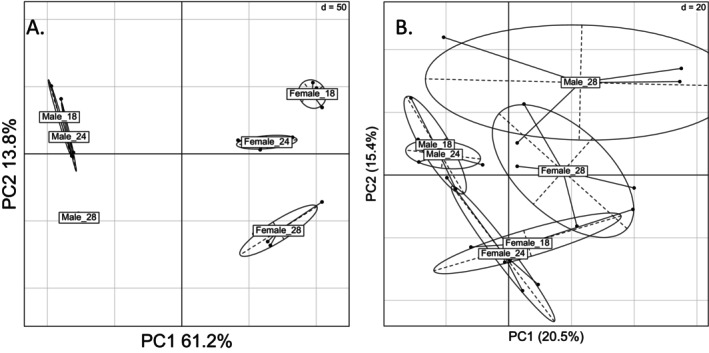
Transcriptomics and Positive metabolomics PCA of temperature and sex. For transcriptomics data PC1 was significantly associated with sex (*p* = 7.30e‐16) and PC2 was significantly associated with temperature (*p* = 2.36e‐7) (A). For metabolomics data (positive mode shown), PC1 was associated with temperature (*p* = 0.037) while sex was associated with PC2 (*p* = 0.0001) (B).

Our GO analysis found that flies raised at warmer temperatures generally had higher levels of genes related to sugar/carbohydrate transmembrane transport (Figure S4A; Table S2) while flies raised at lower temperatures had higher levels of ribosome and proteasome genes (Figure S4B; Table S2). These indicate that environmental temperature is a significant regulator of carbohydrate metabolism and proteasome activity in adulthood. Our PCA of the transcriptome showed clear separation of both sex (PC1) and temperature (PC2) (Figure 4A). Clustering analysis of all transcripts using Euclidean distance found that the sexes clearly separate (Figure S5). The temperatures at which the females were maintained are clearly delineated, but the same pattern is less apparent in males.

We then ran a metabolomics analysis of flies collected at the same time as the transcriptomic analysis. We found 3%–7% of the metabolome changed with adult temperature while less than 1% of metabolites were associated with sex (Table 4, Tables S3 and S4). When we ran our metabolic pathway enrichment analysis, we found 20 metabolic pathways enriched for temperature metabolites, and no pathways enriched for sex differences metabolites (Table S5). Most of these temperature pathways dealt with amino acid metabolism (Figure S6), specifically proline, glutamine, and arginine (Table S5), indicating that amino acid biosynthetic processes are tightly regulated by environmental temperature. Our PCA showed separation of 18°C and 24°C with 28°C, but no separation of 18°C and 24°C (Figure 4B). We then removed 28°C samples from our analysis and found a clearer separation of the temperatures. Sex was also clearly delineated in our metabolomics PCA, even though very few individual metabolites were associated with sex.

**TABLE 4:** Numbers of transcripts and metabolites associated with temperature, sex, and their interaction.

Factor	Genes	Metabolites (+ ion)	Metabolites (− ion)
Temperature	2984 (30.8%)	225 (7.3%)	106 (3.3%)
Sex	3763 (38.8%)	24 (0.8%)	1 (0.03%)
Temperature × Sex	1576 (16.3%)	3 (0.1%)	9 (0.3%)

## 4 Discussion

The *Drosophila* metabolome and transcriptome are both highly influenced by the temperature in which the animals reside, consistent with decades of previously published work; we found that lower temperatures led to increased lifespan in both males and females. We found a temperature by sex interaction in which males were shorter lived at the warm and middle temperature but longer lived at the cool temperature. To discover molecular changes that might explain this interaction, we looked specifically at our transcriptomic and metabolomic analysis for specific interaction genes and metabolites. We found that 16% of genes showed a temperature by sex interaction (Table 2), and these genes might help explain the same interaction that is seen in our longevity analysis. There appear to be strong patterns of increased gene expression in many genes in females at warmer but not standard and low temperature. This sex difference in temperature regulated longevity is something that is not often reported but does exist. Previous work in *D. simulans* suggests that warmer temperature has a more detrimental effect on male lifespan as compared to female (Parsons 1977), just as we see in this study with *D. melanogaster* . Broadly, little is known about sex‐by‐temperature interactions in longevity, presenting a critical knowledge gap. Our data presented here highlight the importance of treating sex as a biological variable, as both our transcriptomic and metabolomic datasets were clearly separated based on sex (Figure 4), and sex had clear effects on the overall lifespan of our flies. Future studies would be well advised to account for sex to ensure these biologically relevant effects are captured.

### 4.1 Turandot Genes in Lifespan Extension

One group of genes that we found to be consistently upregulated in response to increased temperature, as well as showing strong temperature by sex interactions, was the *tot* genes. The *tot* gene family comprises eight *Drosophila‐*specific genes that function in the fruit fly Jak/Stat pathway (Agaisse and Perrimon 2004). Previous research has shown that the entire family of genes is significantly upregulated in response to stress (Ekengren and Hultmark 2001), including heat shock, with a majority of research on the *tot* genes also involved in fly immunity. Unlike the typically studied heat shock proteins, the *tot* genes are secreted into the hemolymph in response to stress (Ekengren and Hultmark 2001). In addition, very young flies (2–3 day adults) appear to show very low *tot* gene expression, with only *totB* and *totC* being detectable by RT‐PCR (Ekengren and Hultmark 2001).

*totA* has previously been shown to be upregulated with age, and *totA* overexpression leads to improved heat stress resistance in female flies (Ekengren et al. 2001). We found all the *tot* genes measured in our study were increased in response to higher temperature (Figure 3), even though this temperature would not be considered as high as a typical “heat shock”. From these results, we hypothesized that knockdowns of the *tot* genes would lead to increased lifespan at warm temperatures with no effect at cooler temperatures. Surprisingly, we observed lifespan changes at our coolest temperature for all *tot* mutants used, highlighting the importance of these genes in regulating lifespan.

### 4.2 Mammalian Orthologs of Turandot Genes

Although the Turandot genes themselves lack direct sequence orthologs in vertebrates, their functional context—transcriptional regulation by the JAK/STAT pathway and secretion into systemic circulation—draws a compelling parallel to mammalian biology. In mammals, the JAK/STAT pathway is a central regulator of cytokine production and the inflammatory response. Chronic activation of JAK/STAT signaling is a hallmark of aging, often termed “inflammaging,” and is a primary driver of the Senescence‐Associated Secretory Phenotype (Bina and Zeidler 2009; Xu et al. 2015; Yan et al. 2018). Much like the *tot* genes in flies, mammalian pro‐inflammatory cytokines (e.g., IL‐6) are secreted systemically and increase with age and stress.

Recent mammalian studies demonstrate that dampening this pathway yields longevity benefits; for instance, treatment with JAK inhibitors (e.g., Ruxolitinib) reduces inflammation, alleviates frailty, and improves physical function in aged mice (Xu et al. 2015). Our finding that *tot* knockdown extends lifespan suggests that Drosophila *tot* genes may function as the evolutionary/functional equivalent of mammalian pro‐inflammatory cytokines. Thus, the longevity benefit we observe upon *tot* reduction potentially stems from a reduction in “sterile inflammation” or the metabolic cost of constitutive immune activation, mirroring the benefits of JAK/STAT inhibition in mammals. Future work evaluating the activity of Turandot proteins, beyond the level of RNA abundance, is warranted to empirically evaluate this hypothesis.

### 4.3 The Interaction of Sex and the Turandot Genes

We found interesting sex‐specific patterns when knocking down *tot* genes across all temperatures. Knocking down *totA*, the most studied of all the *tot* genes, increased longevity in both sexes at all temperatures. However, knocking down *totC* increased lifespan in females, but decreased it in males. Eliminating *totZ* expression had an opposite effect, increasing longevity in males but decreasing it in females. We should interpret this difference cautiously, however, as *totZ* was studied in a different genetic background than either of the RNAi knockdowns. These results suggest that the *tot* family of genes may be “aging” genes and not necessarily temperature‐dependent genes, as we had expected to find that knockdown of the genes would increase lifespan at warmer temperatures with no or little effect at low temperatures due to their low expression profile. While the *tot* genes are a family of duplicated genes, they have overall low sequence homology (Ekengren and Hultmark 2001). Potentially, this divergence in gene sequences may account for the opposite sex‐specific effects we see between *totC* and *totZ*. It is also notable that “omics” analyses were conducted on whole fly bodies, and our knockdowns were not tissue‐specific. This represents a key limitation of our study as the contribution of individual organ systems cannot be determined, which may partially obscure mechanisms for the sex effects we observed in our survival studies. Organ system specific effects of these genes still need to be investigated to more completely understand their role in aging.

### 4.4 Inconsistent Effect of Temperature on Heat Shock Proteins

We were also interested in determining if the more commonly studied heat shock proteins, *hsps*, were differently regulated at the various temperatures, even if our employed temperatures may not represent a major “heat shock”. *hsps* have previously been shown to have strong individual effects on lifespan in *Drosophila* (Tower 2011). We found significant variation in the *hsps* with temperature and often observed opposite patterns than would be expected. For example, *hsp68* overexpression has previously been shown to increase lifespan (Wang et al. 2003); however, we found the highest expression was in the warm, short‐lived flies (Figure S2), though the effect was much stronger in males than females. Our results suggest that we might not fully grasp the roles of *hsp* expression in aging at physiologically “normal” temperatures. In addition, our samples were taken at a much later life stage than most previous expression data in flies, which might explain part of the incongruence in our data and previously published results. Taken together, the heat shock proteins were less changed in response to temperature compared to the Turandot genes, which is similar to previous results seen in *D. simulans* (Manenti et al. 2018).

### 4.5 Influence of Sex on the Transcriptome and the Metabolome

While the overall goal of this study was to determine potential molecular underpinnings of temperature mediated lifespan extension, our largest gene expression differences were found with respect to sex. This result is unsurprising as sex has been shown to cause significant variation in all levels of “omic” variation (e.g., Chang et al. 2011; Hoffman et al. 2014). We utilized fly whole bodies which includes the sex organs which will also lead to strong sex differences in gene and metabolite regulation. We did not find a strong signal of sex in the individual metabolites analysis, as previous studies have reported (e.g., Hoffman et al. 2014; Zhou et al. 2020), though one was seen in the entire metabolomic PCA. Potentially, the later ages at which the metabolome was analyzed in these experiments led to a smaller sex effect; however, as the transcriptomic response to sex was still large at the middle age used, it is puzzling that our metabolomic study failed to show a similar pattern. Another explanation, is that the sex effects for each metabolite are relatively small, but when combined in the entire metabolome, they show a strong signal. Overall, our transcriptomics analysis found many more genes associated with either sex or temperature as compared to individual metabolites, and temperature was more clearly delineated in our transcriptomic as compared to metabolomic analysis.

In conclusion, we identify the Turandot gene family as a novel, temperature‐sensitive influencer of longevity. Our results support a model where chronic activation of stress/immune pathways (JAK/STAT signaling) limits lifespan, similar to the detrimental effects of chronic inflammation in mammals. By identifying these “costly” defense genes, our work lays the foundation for targeting conserved inflammatory pathways to decouple environmental stress from organismal aging.

## Author Contributions

J.M.H. collected data, conducted formal analysis, and took the lead in writing the manuscript. M.P.F. assisted in data collection and conducted formal analysis. A.T.L. assisted in manuscript writing. J.M.H., S.N.A., and L.Y.S. conceived the study, designed experiments, secured funding, and supervised overall direction. All authors contributed critical feedback that shaped the study and manuscript.

## Funding

This work was partially supported by NIH grants R00AG059920 to J.M.H. and AG048264 and AG057734 to L.Y.S. A.T.L. is supported by the Eunice Kennedy Shriver National Institute of Child Health & Human Development of the National Institutes of Health under award number T32HD071866.

## Conflicts of Interest

The authors declare no conflicts of interest.

## Supporting information


**Figure S1:** Transcript levels of five commonly studied “aging” genes in Drosophila. Each dot represents a sample, females are shown in red, and males in blue. Note InR, S6, and Ribosomal protein S6 are all significantly negatively associated with temperature.
**Figure S2:** Pairwise comparisons of transcriptomic data. Volcano plots for male flies maintained at 18°C relative to 28°C (A) and female flies maintained at 18°C relative to 28°C. Turnadot, heat shock protein, and “aging genes” (see Figure S2) are highlighted.
**Figure S3:** Heat shock protein transcripts across temperature and sex. A–G show significant changes with temperature while H–K do not. Each dot represents a sample, females are shown in red, and males in blue.
**Figure S4:**. Gene ontology. Terms associated with differently expressed genes with elevated as temperature increases (A) or reduced abundance as temperature increases (B). Terms were restricted to those that had 10–50 genes in the category as detailed in the methods section. BP, biological processes; CC, cellular component; MF, molecular function.
**Figure S5:** Heatmap of all transcripts. Data have been log transformed and scaled for easier visual representation.
**Figure S6:** Top 10 metabolic pathways most significantly enriched for temperature metabolites.

**Table S1:** Transcriptomic *p*‐values from linear model.
**Table S2:** GO analysis for transcripts associated with temperature.
**Table S3:** Positive ion mode metabolomic *p*‐values from linear model.
**Table S4:** Negative ion mode metabolomic *p*‐values from linear model.
**Table S5:** Metabolic pathway enrichment for metabolites associated with temperature.

## Data Availability

The sequencing data generated from these experiments has been deposited at the Gene Expression Omnibus and is publicly accessible (accession: GSE229837). All data used to generate the statistical analyses and figures in this manuscript are available from the corresponding author upon reasonable request.
