# Supplementary material for: Sex‐Specific Regulation of the Turandot Gene Family Modulates Temperature‐Dependent Lifespan in Drosophila melanogaster
Source: Aging Cell. 2026 May 29;25(6):e70564. doi: 10.1111/acel.70564 (PMC13239812; doi:10.1111/acel.70564)

**Figure S1. Transcript levels of five commonly studied “aging” genes in Drosophila.** Each dot represents a sample, females are shown in red, and males in blue. Note InR, S6, and Ribosomal protein S6 are all significantly negatively associated with temperature.

**
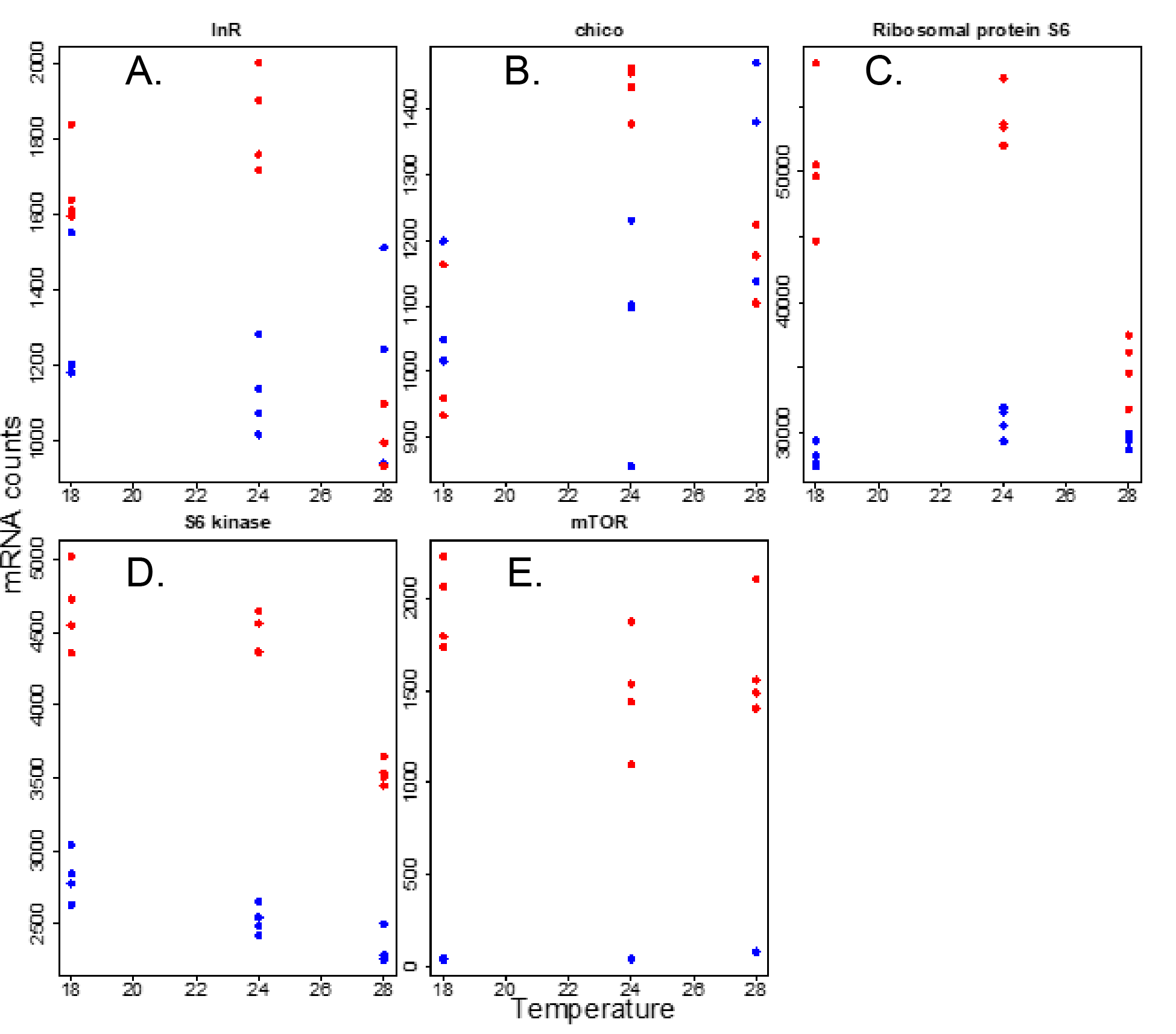
**

**Figure S2. Pairwise comparisons of transcriptomic data.** Volcano plots for male flies maintained at 18°C relative to 28°C (A) and female flies maintained at 18°C relative to 28°C. Turnadot, heat shock protein, and “aging genes” (see Figure S2) are highlighted.

**
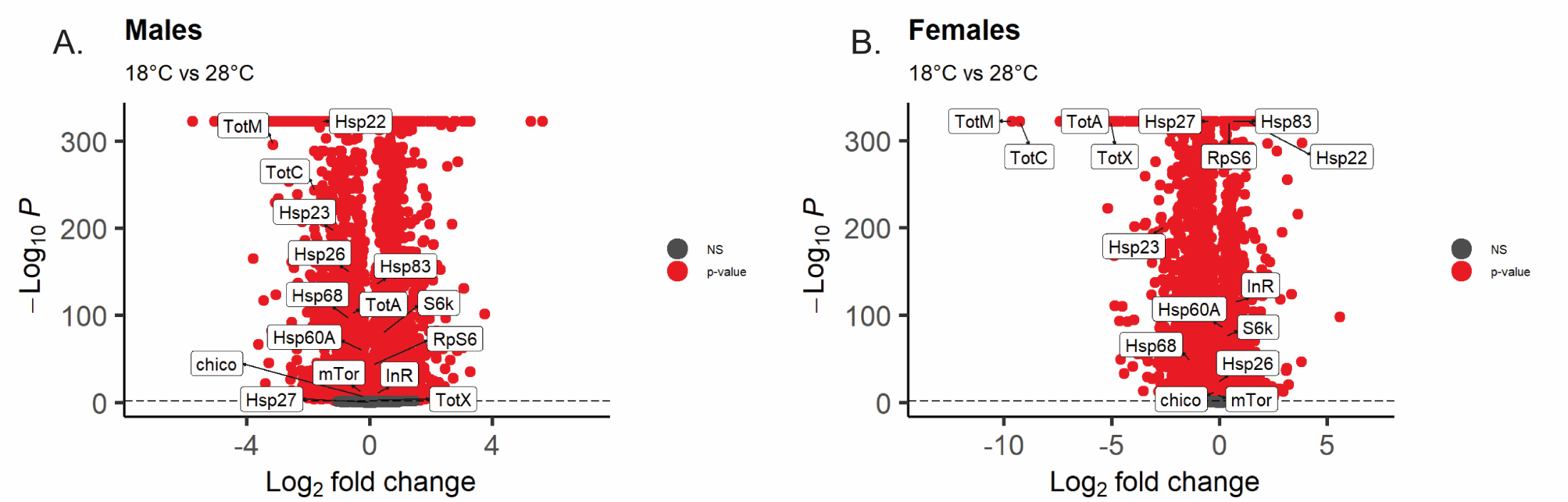
**

**Figure S3. Heat shock protein transcripts across temperature and sex**. A-G show significant changes with temperature while H-K do not. Each dot represents a sample, females are shown in red, and males in blue.

**
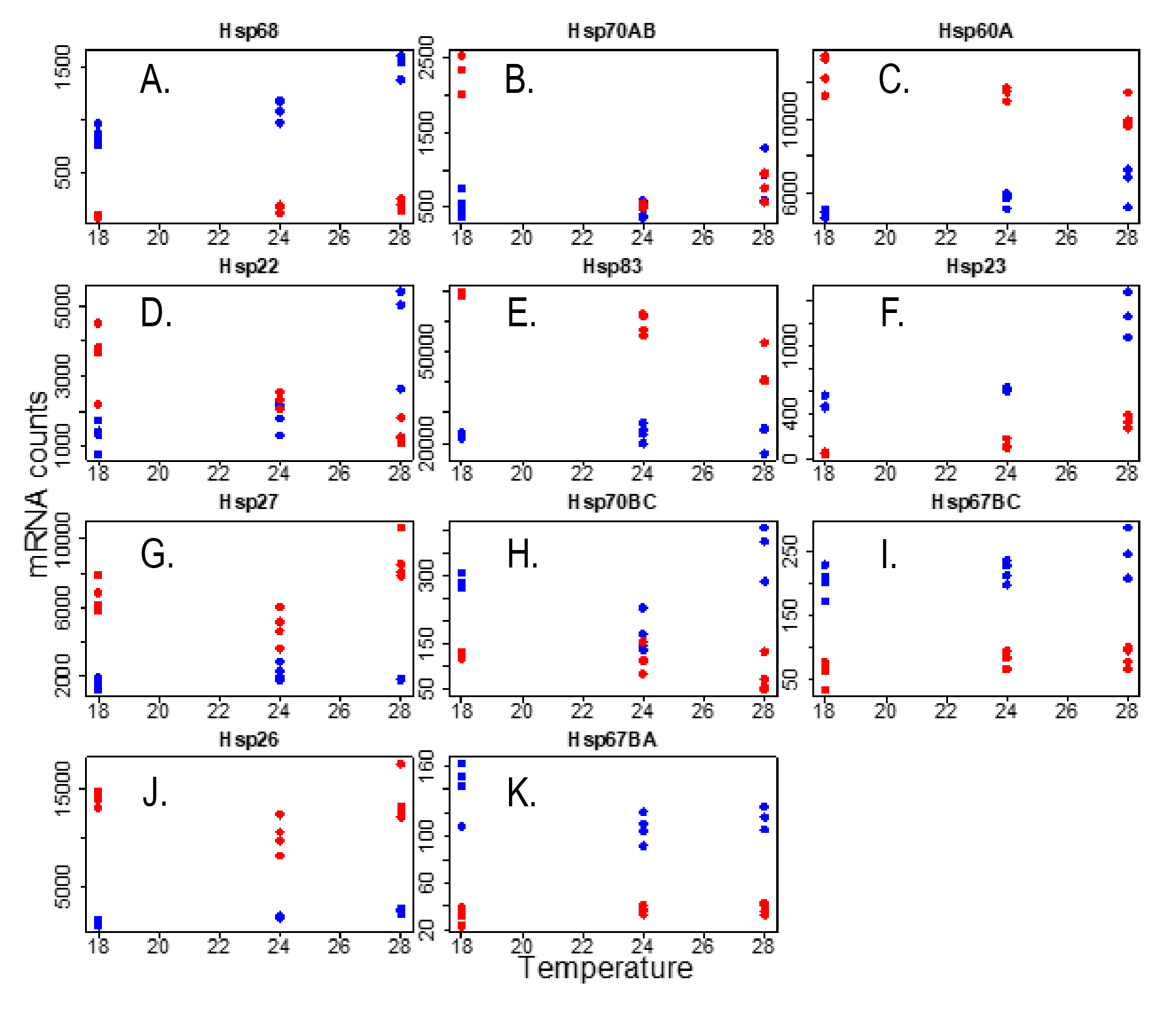
**

**Figure S4**. **Gene ontology.** Terms associated with differently expressed genes with elevated as temperature increases (A) or reduced abundance as temperature increases (B). Terms were restricted to those that had 10-50 genes in the category as detailed in the methods section. BP -biological processes, MF - Molecular Function, CC - Cellular Component.

**
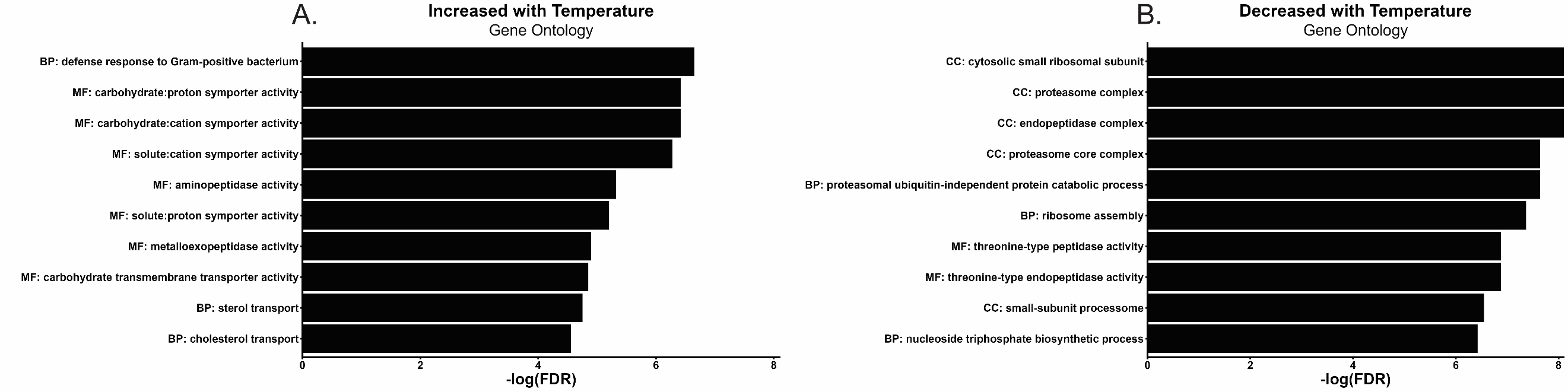
**

**Figure S5. Heatmap of all transcripts.** Data have been log transformed and scaled for easier visual representation.

**
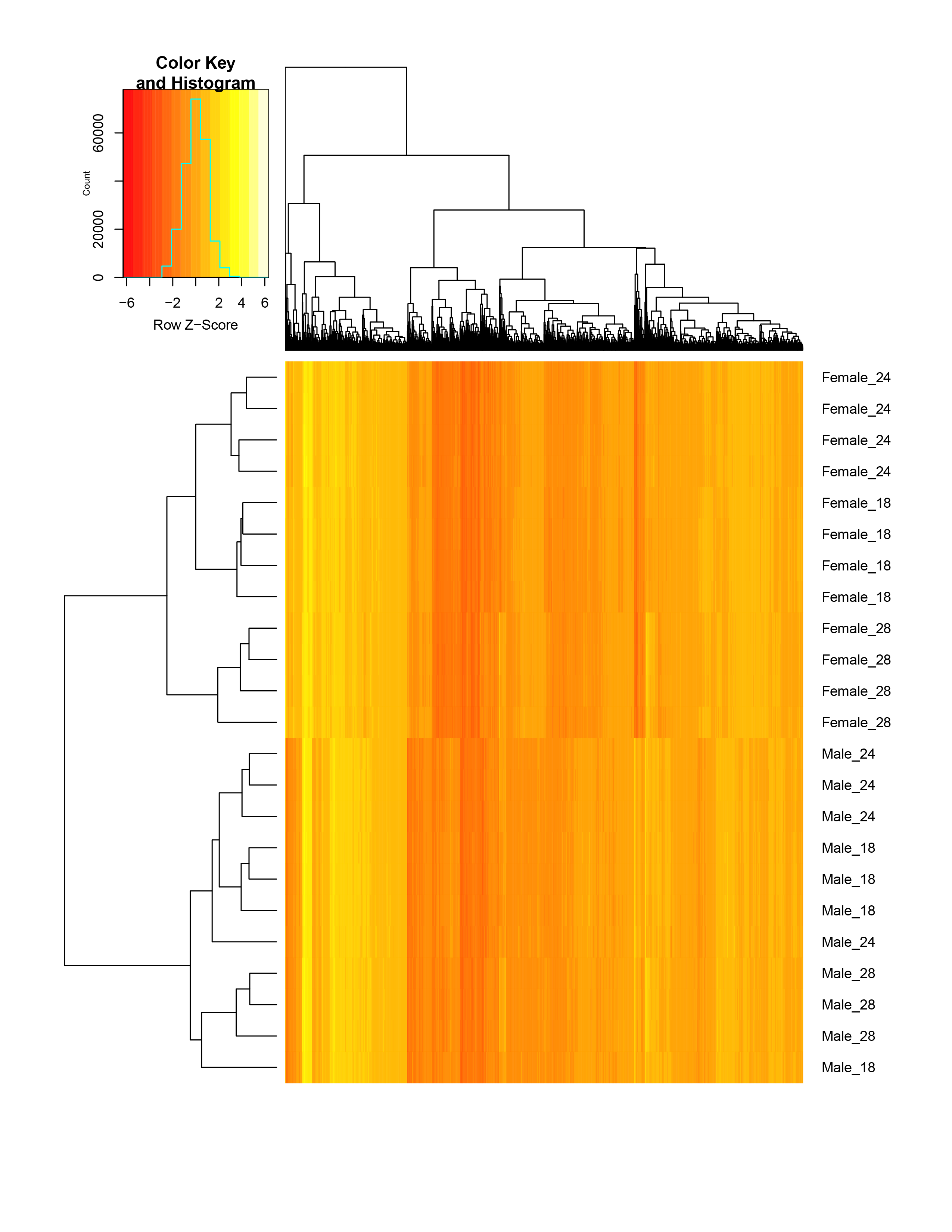
**

**Figure S6.** Top 10 metabolic pathways most significantly enriched for temperature metabolites.


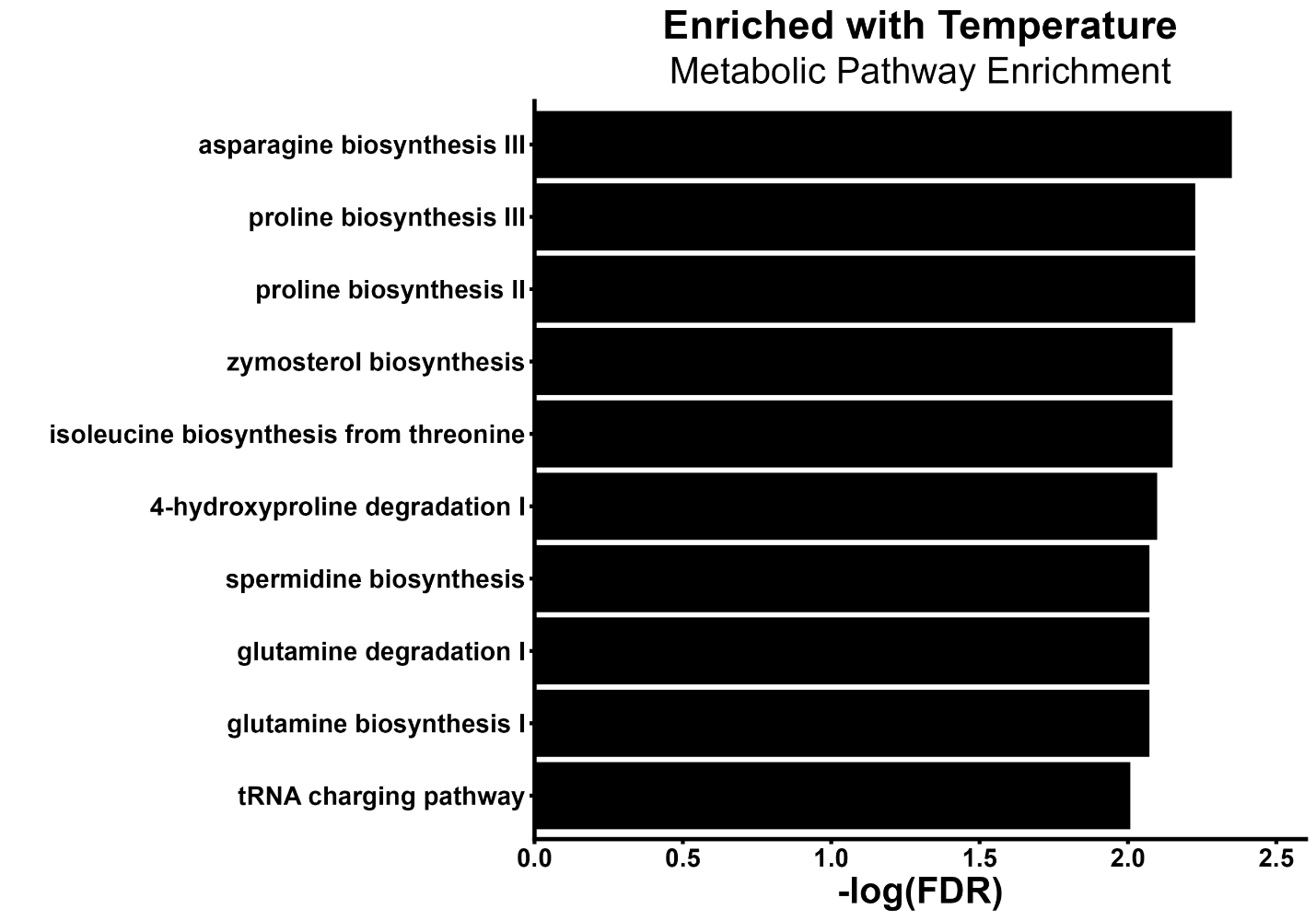

Supplement: Supplementary file 1 — Figure S1: Transcript levels of five commonly studied “aging” genes in Drosophila. Each dot represents a sample, females are shown in red, and males in blue. Note InR, S6, and Ribosomal protein S6 are all significantly negatively associated with temperature. Figure S2: Pairwise comparisons of transcriptomic data. Volcano plots for male flies maintained at 18°C relative to 28°C (A) and female flies maintained at 18°C relative to 28°C. Turnadot, heat shock protein, and “aging genes” (see Figure S2) are highlighted. Figure S3: Heat shock protein transcripts across temperature and sex. A–G show significant changes with temperature while H–K do not. Each dot represents a sample, females are shown in red, and males in blue. Figure S4:. Gene ontology. Terms associated with differently expressed genes with elevated as temperature increases (A) or reduced abundance as temperature increases (B). Terms were restricted to those that had 10–50 genes in the category as detailed in the methods section. BP, biological processes; CC, cellular component; MF, molecular function. Figure S5: Heatmap of all transcripts. Data have been log transformed and scaled for easier visual representation. Figure S6: Top 10 metabolic pathways most significantly enriched for temperature metabolites. [file ACEL-25-e70564-s001.docx]
